# Supplementary material for: Hippocampal and cortical mechanisms at retrieval explain variability in episodic remembering in older adults
Source: eLife. 2020 May 29;9:e55335. doi: 10.7554/eLife.55335 (PMC7259949; doi:10.7554/eLife.55335)
Supplement: Supplementary file 1. — Supplementary file 1a. Neuropsychological test battery performance. Supplementary file 1b. Reaction time (ms) and trial counts as a function of trial type. Supplementary file 1c. Summary of model parameters for mixed effects models. Supplementary file 1d. Summary of linear and logistic mixed effects model results when item miss trials are excluded. Supplementary file 1e. Summary of linear and logistic mixed effects models examining effects of stimulus category (face, place) on relationships between neural variables and behavioural variables. Supplementary file 1f. Summary of linear mixed effects models examining effects of stimulus category (face, place) on relationships between hippocampal activity and cortical reinstatement. Supplementary file 1g. Analysis of head motion and its effects on key dependent variables of interest. Supplementary file 1h. Summary of hierarchical regression analysis predicting associative d’. Supplementary file 1i. Summary of regression analyses examining the relationship between hippocampal subfield activity during associative retrieval (associative hit - CR) and associative memory. [file elife-55335-supp1.docx]

**Supplementary File 1a. Neuropsychological test battery performance.**

| Measure | Mean (SD) | Range |
| --- | --- | --- |
| WTAR | 42.53 (4.31) | 29 – 50 |
| WASI Vocabulary | 72.01 (4.39) | 51 – 78 |
| WASI Similarities | 39.68 (3.22) | 28 – 46 |
| WASI Matrix Reasoning | 26.31 (3.09) | 17 – 31 |
| WASI Block Design | 43.35 (11.90) | 16 – 68 |
| Boston Naming Test | 28.95 (1.35) | 24 – 30 |
| Category Fluency (animals) | 25.83 (5.56) | 12 – 42 |
| Digit Span Forward | 10.70 (2.23) | 6 – 16 |
| Digit Span Backward | 7.92 (2.51) | 3 – 14 |
| Trails A (s) | 32.13 (10.69) | 15 – 65 |
| Trails B (s) | 66.05 (21.12) | 33 – 150 |
| HVLT Immediate Recall ^a^ | 28.74 (4.03) | 14 – 35 |
| HVLT Delayed Recall | 10.49 (1.68) | 5 – 12 |
| HVLT Recognition | 11.01 (1.07) | 8 – 12 |
| BVMT Immediate Recall ^a^ | 24.42 (6.50) | 8 – 36 |
| BVMT Delayed Recall | 9.80 (2.16) | 5 – 12 |
| BVMT Recognition | 5.84 (0.44) | 4 – 6 |
| Logical Memory Immediate Recall | 50.02 (7.59) | 30 – 64 |
| Logical Memory Delayed Recall | 32.04 (6.16) | 18 – 44 |
| Logical Memory Recognition | 27.28 (2.04) | 21 – 30 |

^a^ Sum over three learning trials. WTAR = Wechsler Test of Adult Reading; WASI = Wechsler Abbreviated Scale of Intelligence; BVMT-R = Brief Visuospatial Memory Test-Revised; HVLT-R = Hopkins Verbal Learning Test-Revised.

**Supplementary File 1b. Reaction time (ms) and trial counts as a function of trial type.**

| **Trial Type** | **Median RT (SD)** | **Mean # Trials (SD)** |
| --- | --- | --- |
| Associative Hit^a^  Associative Miss^a^  Item Only Hit^a^  Item Miss^a^  Correct Rejection^a^  Associative Hit^b^ _Face_  Associative Hit^b^ _Place_ | 2382 (421)  2939 (553)  3198 (600)  2689 (568)  2095 (491)  2271 (497)  2548 (413) | 70.15 (19.32)  15.13 (10.35)  13.74 (13.22)  13.65 (9.35)  23.08 (5.31)  39.06 (10.19)  31.09 (10.46) |

^a^ RT varied as a function of response type, such that associative hits were faster than associative misses (*t*(99)=12.09, *p* < 10^-16^), item only hits (*t*(99)=13.80, *p* < 10^-16^), item misses (*t*(99)=4.99, *p* < 10^-6^), and were slower than correct rejections (*t*(99)=2.81, *p* < .01). ^b^Associative hit RT was significantly faster for face trials than place trials (*t*(99)=8.40, *p* < 10^13^).

**Supplementary File 1c. Summary of model parameters for mixed effects models.**

| **IV interest** | **IV nuisance** | **DV** |
| --- | --- | --- |
| Hippocampal Activity | Age, Category | Associative Hit  Exemplar Specific Hit  RT |
| Category-Level Reinstatement Strength (logits) | Age, Category, Encoding Strength (logits), ROI Univariate Activity^a^ | Associative Hit  Exemplar Specific Hit  RT |
| Category-Level Reinstatement Strength (ERS) | Age, Category, Encoding Strength (logits), ROI Univariate Activity^b^ | Associative Hit  Exemplar Specific Hit  RT |
| Event-Level Reinstatement Strength (ERS) | Age, Category, ROI Univariate Activity^b^, Category Reinstatement (ERS) | Associative Hit  Exemplar Specific Hit  RT |
| Hippocampal Activity | Age, Category, Encoding Strength (logits) | Category Reinstatement Strength (logits) |
| Hippocampal Activity | Age, Category, Encoding Strength (logits) | Category Reinstatement Strength (ERS) |
| Hippocampal Activity | Age, Category, Category Reinstatement Strength (ERS) | Event-Level Reinstatement Strength (ERS) |

*Note:* All models include a random intercept for subject and a random slope for the IV of interest. ^a^ = Univariate activity in top 500 category-selective voxels in VTC or ANG over which classification analyses were conducted; ^b^ = Univariate activity in whole ROI (VTC or ANG) over which pattern similarity analyses were conducted; ERS = Encoding Retrieval Similarity; ROI = Region of Interest.

**Supplementary File 1d. Summary of linear and logistic mixed effects model results when item miss trials are excluded.**

| **IV** | **DV** | **χ^2^** | **P** |
| --- | --- | --- | --- |
| Category-Level Reinstatement _VTC_ | Associative Hit | 90.25 | 2.1* 10^-21^ |
| Category-Level Reinstatement * Age  Category-Level Reinstatement _ANG_  Category-Level Reinstatement * Age  Event-Level Reinstatement _VTC_  Event-Level Reinstatement *Age  Event-Level Reinstatement _ANG_  Event-Level Reinstatement *Age  Hippocampal Activity  Hippocampal Activity * Age  Hippocampal Activity  Hippocampal Activity * Age  Hippocampal Activity  Hippocampal Activity * Age  Hippocampal Activity  Hippocampal Activity * Age  Hippocampal Activity  Hippocampal Activity * Age | Associative Hit  Associative Hit  Associative Hit  Associative Hit  Category-Level Reinstatement _VTC_  Category-Level Reinstatement _ANG_  Event-Level Reinstatement _VTC_  Event-Level Reinstatement _ANG_ | 4.48  128.15  2.93  1.78  1.30  7.50  0.02  41.16  0.89  35.26  1.67  24.88  0.65  4.55  0.472  0.78  0.478 | 0.034  1.04 * 10^-29^  0.087  0.183  0.254  0.006  0.900  1.40 * 10^-10^  0.345  2.89*10^-9^  0.197  6.10*10^-7^  0.419  0.033  0.492  0.378  0.489 |

Note: Statistics reflect χ^2^ values (degrees of freedom = 1) and p values for model comparison with and without the primary IV of interest. Category-Level Reinstatement = classifier evidence at retrieval (logits). Event-Level Reinstatement = encoding-retrieval similarity. See Supplementary File 1c for a list of all nuisance regressors included in each model.

**Supplementary File 1e. Summary of linear and logistic mixed effects models examining effects of stimulus category (face, place) on relationships between neural variables and behavioural variables.**

| **IV** | **DV** | **χ^2^** | **P** |
| --- | --- | --- | --- |
| Category-Level Reinstatement _VTC_ | Associative Retrieval Accuracy | 29.55 | 5.4 * 10^-8^ |
|  | - Face Trials - Place Trials | 21.89  75.81 | 2.9 *** 10^-6^  3.1 * 10^-18^ |
| Category-Level  Reinstatement _ANG_  Hippocampal Activity | Decision RT _associative hits_   - Face Trials - Place Trials   Associative Retrieval Accuracy   - Face Trials - Place Trials   Decision RT _associative hits_  - Face Trials  - Place Trials  Associative Retrieval Accuracy  Decision RT _associative hits_ | 9.39  36.40  8.46  9.63  93.73  55.84  92.29  45.51  10.43  2.86  0.01 | 0.002  1.6 * 10^-9^  0.004  0.002  3.6 * 10^-22^  7.9 * 10^-14^  7.5 * 10^-22^  1.5 * 10^-11^  0.001  *p =* .091  *p* = .905 |

Note: Statistics reflect χ^2^ values (degrees of freedom = 1) and p values for model comparison with and without the interaction term. When the interaction term was significant, follow-up regression models were conducted within each stimulus category separately. Category-Level Reinstatement = classifier evidence at retrieval (logits).

**Supplementary File 1f. Summary of linear mixed effects models examining effects of stimulus category (face, place) on relationships between hippocampal activity and cortical reinstatement.**

| **IV** | **DV** | **χ^2^** | **p** |
| --- | --- | --- | --- |
| Hippocampal Activity | Category-Level Reinstatement _VTC_ | 235.87 | 3.1 * 10^-42^ |
|  | - Face Trials - Place Trials | 11.96  82.93 | 0.0005  8.5 * 10^-20^ |
| Hippocampal Activity  Hippocampal Activity  Hippocampal Activity | Category-Level Reinstatement _ANG_   - Face Trials - Place Trials   Event-Level Reinstatement _VTC_   - Face Trials - Place Trials   Event-Level Reinstatement _ANG_ | 8.47  28.28  3.15  4.62  0.02  9.13  2.23 | 0.004  1.1 * 10^-7^  0.077  0.032  0.881  0.003  0.135 |

Note: Statistics reflect χ^2^ values (degrees of freedom = 1) and p values for model comparison with and without the interaction term. When the interaction term was significant, follow-up regression models were conducted within each stimulus category separately. Category-Level Reinstatement = classifier evidence at retrieval (logits). Event-Level Reinstatement = encoding-retrieval similarity.

**Supplementary File 1g. Analysis of head motion and its effects on key dependent variables of interest.**

| **IV** | **DV** | **β** | **p** |
| --- | --- | --- | --- |
| Age  Sex  Education | Head Motion _study_ | 0.19  -0.30  0.03 | 0.053  0.153  0.762 |
|  |  |  |  |
| Age  Sex  Education | Head Motion _test_ | 0.15  -0.29  0.05 | 0.139  0.159  0.611 |
|  |  |  |  |
| Head Motion _study_  Head Motion _study_ | Encoding Strength _VTC_  Encoding Strength _ANG_ | -0.48  -0.34 | 4.2*10-7  0.001 |
|  |  |  |  |
| Head Motion _test_  Head Motion _test_  Head Motion _mean_  Head Motion _mean_  Head Motion _test_ | Category-Level Reinstatement _VTC_  Category-Level Reinstatement _ANG_  Event-Level  Reinstatement _VTC_  Event-Level  Reinstatement _ANG_  Hippocampal Activity | -0.06  -0.19  -0.08  -0.04  -0.16 | 0.521  0.056  0.417  0.652  0.105 |

*Note*. Head motion is computed as mean framewise displacement, separately for study and test runs. Encoding Strength = classifier evidence at encoding (logits); Category-Level Reinstatement = classifier evidence at retrieval (logits); Event-Level Reinstatement = encoding-retrieval similarity. SE = standard error; VTC = ventral temporal cortex; ANG = angular gyrus.

**Supplementary File 1h. Summary of hierarchical regression analysis predicting associative *d’*.**

|  | **Variable** | | **β** | **SE** | **p** | **Adjusted R^2^** |
| --- | --- | --- | --- | --- | --- | --- |
| Step 1 | Age | | -0.324 | 0.096 | 0.001*** | 0.096 |
|  |  | |  |  |  |  |
| Step 2 | Age | | -0.283 | 0.090 | 0.001*** | 0.205 |
|  | Hippocampal Activity^a^ | | 0.349 | 0.091 | 0.001*** |  |
|  |  | |  |  |  |  |
| Step 3a | Age | | -0.169 | 0.090 | 0.063~ | 0.295 |
|  | Hippocampal Activity^a^  VTC Reinstatement^ab^ | | 0.284  0.368 | 0.088  0.100 | 0.002**  0.001*** |  |
|  |  | |  |  |  |  |
| Step 3b | Age | | -0.245 | 0.089 | 0.007** | 0.249 |
|  | Hippocampal Activity^a^  ANG Reinstatement^ab^ | | 0.282  0.303 | 0.093  0.117 | 0.003**  0.011* |  |
|  |  | |  |  |  |  |
| Step 4 | Age | | -0.179 | 0.090 | 0.064~ | 0.297 |
| Step 5 | Hippocampal Activity^a^  VTC Reinstatement^ab^  ANG Reinstatement^ab^  Age  Hippocampal Activity^a^ | | 0.262  0.310  0.146  -0.119  0.228 | 0.089  0.113  0.127  0.090  0.088 | 0.004***  0.007***  0.254  0.190  0.012* | 0.336 |
|  | VTC Reinstatement^ab^  Delayed Recall | | 0.312  0.328 | 0.100  0.124 | 0.002**  0.009** |  |
|  |  |  | |  |  |  |

*Note*. ^a^ = adjusted by head motion; ^b^ = adjusted by encoding strength (mean logits across leave-one-run-out-n-fold cross validation); Reinstatement = category-level reinstatement (mean logits across associative hits); SE= standard error; VTC = ventral temporal cortex; ANG = angular gyrus; ~ p < 0.1, * p < 0.05, ** p < .01, *** p < .001 **** p < 10^-5^

**Supplementary File 1i. Summary of regression analyses examining the relationship between hippocampal subfield activity during associative retrieval (associative hit - CR) and associative memory.**

| **IV** | **DV** | **β** | **p** |
| --- | --- | --- | --- |
| DG/CA3_a_  CA_a_  Sub_a_  Head_a_  Tail_a_ | Associative *d’*_b_  Associative *d’*_b_  Associative *d’*_b_  Associative *d’*_b_  Associative *d’*_b_ | 0.25  0.19  0.19  0.30  0.26 | 0.007  0.045  0.044  0.001  0.005 |
| DG/CA3_a_  CA_a_  Sub_a_  Head_a_  Tail_a_ | Exemplar-Specific Recall_b_  Exemplar-Specific Recall_b_  Exemplar-Specific Recall_b_  Exemplar-Specific Recall_b_  Exemplar-Specific Recall_b_ | 0.30  0.27  0.27  0.41  0.26 | 0.002  0.004  0.006  7.5 * 10^-6^  0.007 |

*Note*. ^a^ = adjusted by head motion. ^b^ = adjusted by age. DG = dentate gyrus; Sub = Subiculum.
